# Effect of Nitrogen Source and Inorganic Phosphate Concentration on Methanol Utilization and *PEX* Genes Expression in *Pichia pastoris*

**DOI:** 10.1155/2014/743615

**Published:** 2014-12-24

**Authors:** A. M. Rumjantsev, O. V. Bondareva, M. V. Padkina, E. V. Sambuk

**Affiliations:** Department of Genetics and Biotechnology, St. Petersburg State University, St. Petersburg 199034, Russia

## Abstract

Methylotrophic yeast *Pichia pastoris* has proved to be especially useful for production of various heterologous proteins. In biotechnology it is very important to maintain the balance between high levels of heterologous gene expression and cell viability. Decisive understanding of gene regulation mechanisms is essential for reaching this goal. In this study, we investigated the effect of different nitrogen sources and phosphate concentration in media on methanol utilization. It was shown that expression levels of main genes, which are involved in methanol utilization (*MUT* genes) and in functioning of peroxisomes (*PEX* genes), are maximal when ammonium sulphate is used as a nitrogen source. Expression of these genes is decreased in media with poor nitrogen sources, such as proline. Addition of rapamycin to the media completely removed repression of *AOX1* promoter in media with proline, which allows proposing that Tor-kinase is involved in establishing of nitrogen regulation of this gene. It was also shown that *MUT* genes expression levels get higher, when the phosphate concentration in media is increased.

## 1. Introduction

Microorganisms adaptation to different environment changes is primarily established on transcriptional level. Hence, the expression of most enzyme encoding genes changes dramatically in nutrient deficiency conditions. Yeast* Pichia pastoris* represent the eukaryotic group capable of utilizing methanol as a sole carbon source. Methanol utilization pathway is common in all methylotrophic yeasts and involves several unique enzymes [1]. Alcohol oxidase (Aox EC 1.1.3.13) catalyzes methanol oxidation to formaldehyde. Hydrogen peroxide, which is also produced in this reaction, is degraded by catalase (Cat EC 1.11.1.6) to oxygen and water. Hydrogen peroxide formation is very dangerous for the living cell; thus alcohol oxidase and catalase are sequestered within special organelles—peroxisomes [2]. Proteins involved in peroxisome biogenesis are called peroxines (or PEX proteins) and are encoded by* PEX* genes [3].

A portion of formaldehyde generated by alcohol oxidase leaves peroxisomes and is further oxidized by formaldehyde dehydrogenase (Fld EC 1.2.1.1) and formate dehydrogenase (Fdh EC 1.2.1.2) providing energy for cells.* S*-formylglutathione hydrolase (Fgh EC 3.1.2.12) participates in the detoxication of formaldehyde and regenerates glutathione.

Another portion of formaldehyde is condensed with xylulose 5-phosphate by the third peroxisome enzyme—dihydroxyacetone synthase (Dhas EC 2.2.1.3) resulting in generation of glyceraldehyde 3-phosphate and dihydroxyacetone. These two tricarbonic compounds are further involved in xylulose 5-phosphate regeneration resulting in one novel molecule of glyceraldehyde 3-phosphate for every three cycles [1, 2].

Expression of alcohol oxidase genes is strictly regulated by the type of carbon source presented in the media. In* P. pastoris* cells grown on glucose (or glycerol) alcohol oxidase is not detectable. However, in methanol-grown cells alcohol oxidase levels increase dramatically, compromising up to 30% of total soluble protein [4].

*P. pastoris* alcohol oxidase has two isoforms which are the products of* AOX1* and* AOX2* genes. The coding regions of* AOX1* and* AOX2* genes have more than 90% similarity. But promoter regions are significantly different, and product of* AOX1* gene provides about 90% of alcohol oxidase activity [4]. Regulation of* AOX1* gene expression is established on transcriptional level and consists of two separate mechanisms, providing repression in the presence of various carbon sources and methanol induction. In repressing conditions, while glucose or glycerol are presented in the media,* AOX1* expression is practically not detectable. In derepressing conditions, for example, carbon starvation,* AOX1* gene expression levels increase 1000-fold in comparison with repressing conditions but still compromise only about 2% of expression levels, observed in methanol-grown cells [4, 5]. Such mechanisms of gene regulation by alternative carbon sources are similar for various microorganisms [6]. Due to the tight regulation capability and high activity levels of* AOX1* gene, its promoter is widely used for heterologous protein production in* P. pastoris*.

It is known that transcription of several yeast genes that are involved in carbon utilization also depends on the type of nitrogen source and phosphate concentration in media [7, 8]. This allows the cells to adapt to nutrient limitation by correction of gene expression to optimum levels.

Methylotrophic yeasts are able to utilize vast variety of nitrogen containing compounds. However, for the majority of yeast species glutamine, glutamic acid and ammonium are preferable as nitrogen sources, and they have developed special regulation mechanisms that provide preemptive absorption of these compounds [9]. But if such preferential nitrogen sources are not presented in the media yeast cells switch their metabolic pathways and begin to utilize poor nitrogen sources such as urea and proline.

In previous studies we used a convenient test system containing a promoter of the* AOX1* gene and the* PHO5* gene of* Saccharomyces cerevisiae* as a reporter gene [10].* PHO5* gene encodes yeast acid phosphatase (ACP), which is secreted to the surface of cells. The ACP activity is easily determined by both qualitative and quantitative methods [11]. It was shown that* AOX1* gene expression depends on the type of nitrogen source. The highest ACP activity levels were observed in media with glutamine and ammonium, while decreased levels were observed when glutamic acid and proline were used as a nitrogen source [10].

Genetic regulation of nitrogen source signal transduction is well studied in yeast* S. cerevisiae*. The key role in establishing this regulation is played by serine-threonine kinases Tor (target of rapamycin). Most of eukaryotic organisms have only one gene which encodes Tor-kinase, while in* S. cerevisiae* there are two different kinases—Tor1p and Tor2p [12]. This can be explained by the fact that* S. cerevisiae* genome arose from complete duplication of eight ancestral chromosomes [13]. TORC1 complex is involved in regulation of cell proliferation and regulation of cell growth in different nutrient conditions. TORC2 complex regulates actin cytoskeleton organization and cell polarity but is not involved in regulation of metabolic processes [14]. Tor-kinase is a main target for rapamycin, which completely inhibits its activity [15]. Rapamycin treatment of cells induces processes similar to nitrogen starvation: block of translation, protein degradation, autophagy, glycogen accumulation, sporulation, and arrest of cell cycle [16]. Also rapamycin treatment changes the expression of several hundreds of genes, including the ones involved in carbon metabolism, in a way similar to nitrogen starvation [17].

In* S. cerevisiae* phosphate limitation induces dramatic changes in cell metabolism and modifies expression of large number of genes [18]. A key role in establishing phosphate regulation in* S. cerevisiae* is played by another serine-threonine kinase [19].

In this study, we demonstrated the effect of different nitrogen sources and phosphate concentration in media on expression of genes involved in methanol utilization and peroxisome biogenesis. It was shown that rapamycin treatment influences* AOX1* gene expression in media with different nitrogen sources.

## 2. Materials and Methods

### 2.1. Plasmids

*pPIC9-PAOX1-PHO5* plasmid contains* AOX1* promoter and the* PHO5* gene of* S. cerevisiae*, which encodes yeast acid phosphatase. It was created by cloning of a PCR amplified* PHO5* gene into* pPIC9* vector (Invitrogen) using* BamHI* and* EcoRI* sites [10].

*pPIC9-delSacI-PAOX1-PHO5* and* pPIC9-delNsiI-PAOX1-PHO5* carry 201 bp and 671 bp truncations of* AOX1* promoter. To create these plasmids* pPIC9-PAOX1-PHO5* was cut in* AatII* site and one of the unique sites within* AOX1* promoter* SacI* or* NsiI*. The resulting fragments were blunted using* Pfu*-polymerase and self-ligated [10].

*pPIC9-PAOX2-PHO5* plasmid was created using* pPIC9-PAOX1-PHO5* and contains* PHO5* gene under control of* AOX2* promoter. A PCR amplified* AOX2* promoter fragment was cloned into* pPIC9-PAOX1-PHO5* using* AatII* and* BamHI* restriction sites, so it replaced the* AOX1* promoter.

*pPIC9-delA-PAOX1-PHO5* plasmid contains a 80 bp deletion in* AOX1* promoter.* pPIC9-delB-PAOX1-PHO5* contains a 34 bp deletion,* pPIC9-delC-PAOX1-PHO5* a 160 bp deletion, and* pPIC9-delD-PAOX1-PHO5* a 80 bp deletion. Deletions in four regions of* AOX1* promoter named A, B, C, and D (Figure 4(a)) were generated using site-specific mutagenesis. In the first round of PCR two fragments flanking the desired deletion were amplified. Reverse primer for promoter region upstream of deletion and forward primer for downstream region were designed to contain complementary sequences on their 5′ ends. This allowed combining the fragments together in the second round of PCR. Resulting* AOX1* promoter fragments with desired deletions were cloned using* AatII* and* BamHI* restriction sites into* pPIC9-PAOX1-PHO5* plasmid instead of native promoter.

All plasmids were verified using PCR, restriction, and sequencing analysis.

### 2.2. Strains

*P. pastoris* strains presented in Table 1 were used. All strains were derived from the original* P. pastoris* strain GS115 (*his4*) (Invitrogen). 1-GS115 strain lacks ACP activity. tr2-1-GS115 strain carries a reporter acid phosphatase (ACP)* PHO5* gene of* S. cerevisiae* under the control of* AOX1* gene promoter. It was created by transforming of 1-GS115 strain with* pPIC9-PAOX1-PHO5* vector that was cut in* StuI* site.

tr3-1-GS115* P. pastoris* strain, which carries* PHO5* gene under the control of* AOX2* promoter, was created by transforming of 1-GS115 strain with* pPIC9-PAOX2-PHO5* vector that was cut in* StuI* site.

trΔ*SacI*-1-GS115 and trΔ*NsiI*-1-GS115 strains carry* PHO5* gene under the control of* AOX1* gene promoters that were truncated using* SacI* and* NsiI* restriction sites. These strains were created by transforming of 1-GS115 strain with* pPIC9-delSacI-PAOX1-PHO5* and* pPIC9-delNsiI-PAOX1-PHO5* vectors that were cut in* StuI* site.

trA-1-GS115, trB-1-GS115, trC-1-GS115, and trD-1-GS115 strains carry a* PHO5* reporter gene under the control of* AOX1* promoter with different deletions (Figure 4(a)). These strains were obtained by transforming of 1-GS115 strain with* pPIC9-delA-PAOX1-PHO5*,* pPIC9-delB-PAOX1-PHO5*,* pPIC9-delC-PAOX1-PHO5*, and* pPIC9-delD-PAOX1-PHO5* vectors, respectively.

All strains are capable of methanol utilization, due to the fact that plasmids were integrated into* his4* region.

The bacterial* E. coli* strain DH5*α* [F′phi80d*lac*Z delta (*lac*ZYA_*arg*F) U169* deo*R recA1* end*A1* hsd*R17 (rK– mK+)* pho*A* sup*E44 lambda_*thi*_1* gyr*A96* rel*A1/F′* pro*AB+* lac*IqdeltaM15 Tn*10*(*tetr*)] was used for the construction of plasmids.

### 2.3. Culture Media and Conditions

In this study standard rich YPD and synthetic MP media were used. YPD medium contained 20 g of glucose, 20 g of peptone, and 10 g of yeast extract per 1 L. All variations of MP media contained the following per 1 L: 100 mL of 0,1 M Na-citrate buffer pH 4,5; 0,5 g MgSO_4_ *·*7H_2_O; 0,4 g CaCl_2_; vitamins and trace metals; 20 mg of histidine (for GS115 strain) 1% glycerol or 1% methanol as a sole carbon source. Three modifications of MP media were used: (1) MPN contained ammonium sulphate in concentration 0,46 g/L and KH_2_PO_4_ in concentration 1 g/L, (2) MPP contained proline in concentration 0,46 g/L and KH_2_PO_4_ in concentration 1 g/L, and (3) MPL contained ammonium sulphate in concentration 0,46 g/L and KH_2_PO_4_ in concentration 0,03 g/L.* P. pastoris* cells were grown at 25°C.

LB medium was used to cultivate bacterial strains.* E. coli* strains were grown at 37°C.

### 2.4. Oligonucleotides

All oligonucleotides used in this study are presented in Table 2. Probes for real-time PCR were modified with ROX-BHQ2. The Primer 3 program was used to select primers for real-time PCR (http://primer3.sourceforge.net/).

### 2.5. Molecular Methods

The transformation of bacteria and isolation of plasmid DNA from* E. coli* was carried out in accordance with standard methods [20]. The isolation of DNA and yeast transformation was carried out according to [21, 22]. All plasmids that were used in this work carry* HIS4* gene as a selective marker; consequently media without histidine were used for selection of transformed clones.

PCR was performed according to the protocol of the manufacturer of reagents (Thermoscientific).

During real-time PCR experiments total RNA was isolated from an equal number of cells in each culture according to [23]. RNA was treated with DNase; then cDNA was synthesized by reverse transcription and used as a template for real-time PCR.* ACT1* was used as a reference gene. Real-time PCR was performed using an ANK-32 nucleic acids amplifier (Synthol, Russia) and TaqMan technology as follows: 3 min at 95°C, followed by 40 cycles for 30 s at 95°C and 30 s at 60°C. The annealing temperature for all primers was 60°C. All real-time PCR experiments were set at least in triplicate.

DNA hydrolysis with restriction endonucleases, dephosphorylation of vectors, and DNA ligation were performed using the buffers and conditions recommended by the manufacturer of the enzymes (Thermoscientific). Electrophoresis of DNA and purification of DNA from agarose gels were performed according to [20].

ACP activity was determined qualitatively [11] and quantitatively [24]. The specific activity of ACP was designated as the ratio of the optical density at 410 nm to the density of cell suspension at 550 nm.

Statistical analysis was performed using the Statistic program.

## 3. Results

### 3.1. Effect of Nitrogen Source and Phosphate Concentration on Expression of Genes Involved in Methanol Utilization (*MUT* Genes)

In previous study it was found that type of nitrogen source presented in the medium influences the expression levels of* AOX* and* PpCAT1* genes, which encode enzymes involved in first steps of methanol utilization [10]. In this study we investigated regulation of other enzyme coding genes:* PpDAK*,* PpDHAS*,* PpFLD*, and* PpFGH*. Another group of studied genes consisted of* PpPEX5* and* PpPEX14*, encoding peroxisomal structural proteins, and* PpPEX1* and* PpPEX8* genes encoding proteins, involved in biogenesis and degradation of peroxisomes. Real-time PCR, which enables a quantitative analysis of gene expression levels, was used to investigate the effect of the nitrogen source and phosphate concentration on expression of chosen* MUT* genes.

**Figure 1:**
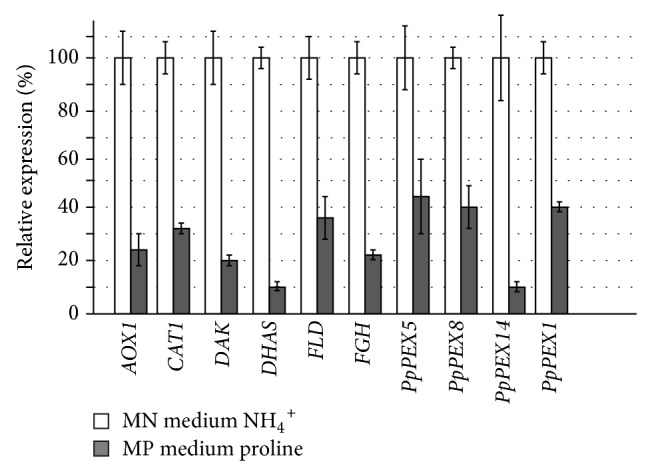
Quantitative analysis of the relative expression levels of* PpDAK*,* PpDHAS*,* PpFLD*, and* PpFGH* and* PpPEX5*,* PpPEX14*,* PpPEX8*, and* PpPEX1* genes depending on the nitrogen source in the medium. MN medium contained ammonium sulphate and 1% methanol. MP medium contained proline and 1% methanol.

To study the effect of the nitrogen source cells of the parental GS115* P. pastoris* strain were grown for 20 hours to the log phase in MPN and MPP media containing methanol as a carbon source. Ammonium sulfate and proline were chosen as nitrogen sources because they demonstrated the most contrasting results in previous experiments [10]. Results of a quantitative analysis of the relative expression levels of* PpDAK*,* PpDHAS*,* PpFLD*, and* PpFGH* and* PpPEX1*,* PpPEX5*,* PpPEX8*, and* PpPEX14* genes depending on the nitrogen source in the media are demonstrated in Figure 1.

The expression of all studied genes in medium with ammonium sulphate was significantly higher than in medium with proline as a nitrogen source. This goes together with the results obtained in previous studies for* AOX* and* PpCAT1* genes. Thus, it was demonstrated that regulation of main* MUT* and* PEX* genes expression depends on the type of nitrogen source in the media and is established at the transcriptional level.

To study the effect of phosphate concentration on expression of* MUT* genes cells of the parental GS115 strain were grown for 20 hours to the log phase in MPN and MPL media containing methanol as a carbon source. Ammonium sulfate was chosen as nitrogen source. KH_2_PO_4_ concentrations used were 0,03 g/L (MPL) and 1 g/L (MPN), because they demonstrated the most contrasting results in previous experiments.

**Figure 2:**
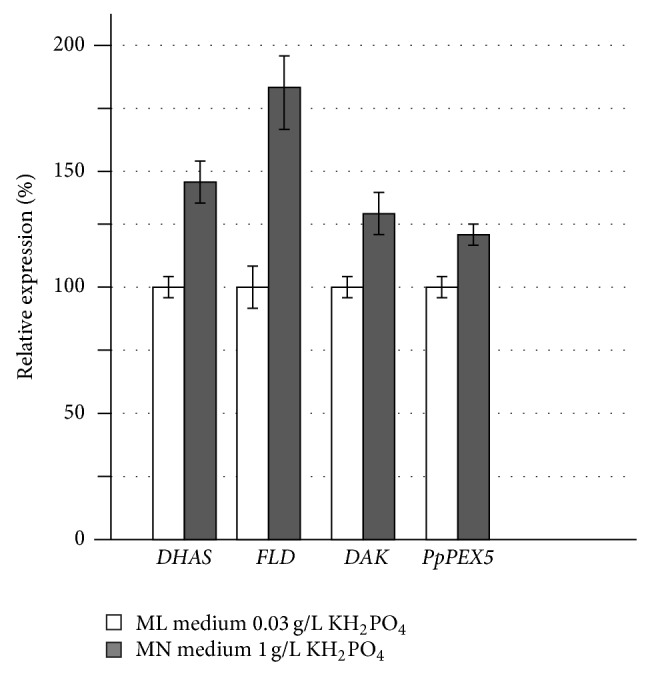
Quantitative analysis of the relative expression levels of* PpDAK*,* PpDHAS*,* PpFLD*, and* PpPEX5* genes depending on KH_2_PO_4_ concentration in the medium. MN media contained 1 g/L KH_2_PO_4_ and 1% methanol. ML media contained 0,03 g/L KH_2_PO_4_ and 1% methanol.

Investigation of* MUT* and* PEX* genes regulation by carbon source [25] and our experiments with nitrogen sources allowed proposing that their activity is controlled by common transcription factors and is regulated in similar way under different nutrient conditions. That is why in this experiment studied genes were limited to* PpDAK*,* PpDAS*, and* PpFLD* genes for* MUT* genes and* PpPEX5* for* PEX* genes. Results of a quantitative analysis of the relative expression levels of* PpDAK*,* PpDAS*,* PpFLD*, and* PpPEX5* genes depending on the phosphate concentration in the medium are shown in Figure 2.

Thus, it was demonstrated that regulation of* PpDAK*,* PpDAS*,* PpFLD*, and* PpPEX5* genes also depends on phosphate concentration in the medium and is established at the transcriptional level.

### 3.2. Effect of Nitrogen Source and Phosphate Concentration on Activity of* AOX1* and* AOX2* Promoters

The protein-coding regions of the* AOX1* and* AOX2* genes for 90% [26] are identical. Thus it is very difficult to analyze their regulation separately using real-time PCR. Creation of convenient reporter systems was essential for solving this problem. tr2-1-GS115* P. pastoris* strain carrying a reporter acid phosphatase (ACP)* PHO5* gene of* S. cerevisiae* under the control of* AOX1* gene promoter was constructed in previous studies [10]. A similar strain tr3-1-GS115 carrying* PHO5* gene under control of* AOX2* promoter was generated in this study.

**Figure 3:**
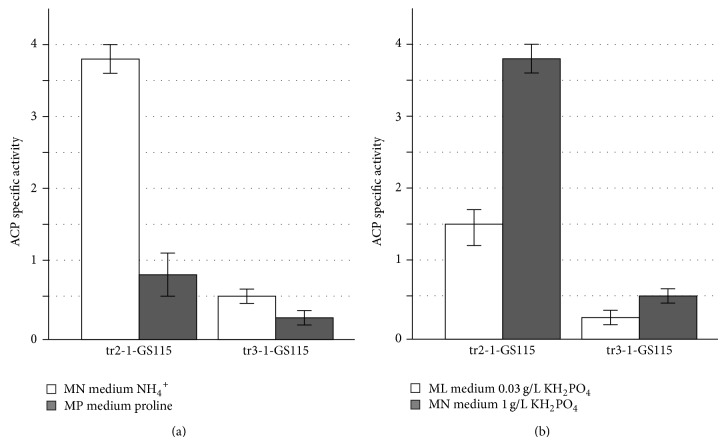
ACP specific activity of* P. pastoris* strains tr2-1-GS115 (*phox PAOX1-PHO5 HIS4*) and tr3-1-GS115 (*phox PAOX2-PHO5 HIS4*) grown in different media: (a) cells were grown in media with different nitrogen sources (MN with ammonium sulphate and MP with proline); (b) cells were grown in media with different concentrations of KH_2_PO_4_ (MN with 1 g/L and in ML with 0,03 g/L).

To investigate the effect of nitrogen source on the activity of* AOX2* promoter tr2-1-GS115 and tr3-1-GS115* P. pastoris* strains were grown for 40 hours to the stationary phase in MPN and MPP media containing methanol as a carbon source. tr2-1-GS115 strain carrying* PHO5* gene under the control of* AOX1* gene promoter was used as a control. The specific activity of ACP was measured in the yeast culture (Figure 3(a)).

The figure shows that the expression of a reporter gene in both strains depends on nitrogen source. The highest ACP activity for both strains was detected in media with ammonium sulfate. ACP activity was significantly lower in media with poor nitrogen source proline. These data suggest that although* AOX1* and* AOX2* promoter regions are significantly different, they are regulated by nitrogen source in similar way.

To study the influence of phosphate concentration on the expression of the* AOX1* and* AOX2* genes, tr2-1-GS115 and tr3-1-GS115* P. pastoris* strains were grown for 40 hours to the stationary phase in MPN and MPL media containing different concentrations of KH_2_PO_4_ (0,03 g/L and 1 g/L, resp.). These concentrations showed most contrast results in primary experiments and they are also similar to the ones that are used in studies of phosphate regulation in* S. cerevisiae* [26]. The specific activity of ACP was measured in the yeast culture (Figure 3(b)).

The figure shows that expression of a reporter gene in both strains depends on phosphate concentration in medium. The highest ACP activity for both tr2-1-GS115 and tr3-1-GS115 strains was detected in media with high phosphate concentration (1 g/L). ACP activity was almost two times lower in media with only 0,03 mg/L of KH_2_PO_4_. These data suggest that activity of* AOX1* and* AOX2* promoters depends on phosphate concentration in medium and is regulated in similar way.

### 3.3. Deletion Analysis of* AOX1* Promoter

In previous studies deletion analysis of the promoter was carried out to identify* AOX1* promoter regions involved in nitrogen regulation. In trΔ*Nsi*I-1-GS115 strain, containing* PHO5* gene under the control of* AOX1* promoter carrying a 671-nucleotide truncation, regulation of expression ACP activity by nitrogen source still remained [10].

**Figure 4:**
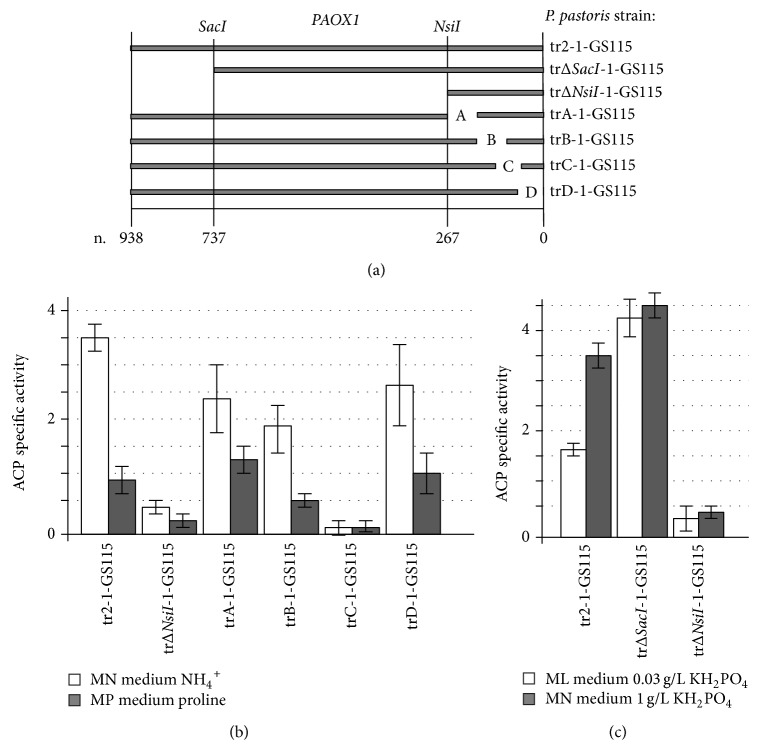
(a) Strains created in this study carry* PHO5* reporter gene under control of different variants of* AOX1* promoter: tr2-1-GS115 carries a native promoter, trΔ*SacI*-1-GS115—a variant truncated in* SacI* site (−737), trΔ*NsiI*-1-GS115—a variant truncated in* NsiI* site (−267), trA-1-GS115 carries* AOX1* promoter with deletion from −296 to −216, trB-1-GS115 from −222 to −188, trC-1-GS115 from −205 to −45, and trD-1-GS115 from −80 to −0 nucleotides. (b) ACP specific activity of* P. pastoris* strains trA-1-GS115, trB-1-GS115, trC-1-GS115, trD-1-GS115, and tr2-1-GS115 in media with different nitrogen sources (MN with ammonium sulphate and MP with proline). (c) ACP specific activity of* P. pastoris* strains trΔ*SacI*-1-GS115, trΔ*NsiI*-1-GS115, andtr2-1-GS115 in media with different concentrations of KH_2_PO_4_ (MN with 1 g/L and in ML with 0,03 g/L).

Thus a 267-nucleotide region was found to be sufficient to establish* AOX1* promoter regulation by the nitrogen source. In this study this region was covered with a series of four deletions: A from −296 to −216, B from −222 to −188, C from −205 to −45, and D from −80 to −0.* P. pastoris* strains trA-1-GS115, trB-1-GS115, trC-1-GS115, and trD-1-GS115 which carry a reporter* PHO5* gene under the control of* AOX1* gene promoter with desired deletions were obtained (Figure 4(a)). These strains were grown for 40 hours to the stationary phase in MPN and MPP media containing methanol as a carbon source. tr2-1-GS115 strain carrying* PHO5* gene under the control of native* AOX1* promoter was used as a control.

Figure 4(b) shows that for trA-1-GS115, trB-1-GS115, and trD-1-GS115 strains the highest ACP specific activity was observed in medium with ammonium sulfate. The ACP specific activity of all three strains was significantly lower in medium with poor nitrogen source proline, suggesting that these deletions do not influence regulation of* AOX1* promoter by nitrogen source. In the case of trC-1-GS115 strain ACP specific activity was extremely low and did not allow us to get reliable results. This can be explained by that fact that the deletion in C fragment of* AOX1* gene carried by this strain contains the main binding site for Mxr1 protein which is known to be the main regulator of* MUT* genes and is essential for methanol induction [27].

To study if the effect of phosphate concentration on regulation of* AOX1* promoter is affected by different deletions we used trΔ*SacI*-1-GS115 and trΔ*NsiI*-1-GS115 strains that were created previously. These strains contain 207- and 671-nucleotide truncations in* AOX1* promoter, respectively (Figure 4(a)). They were grown for 40 hours to the stationary phase in MPN and MPL media containing methanol as a carbon source. tr2-1-GS115 strain carrying* PHO5* gene under the control of native* AOX1* promoter was used as a control.

Figure 4(c) shows that the overall ACP specific activity of trΔ*SacI*-1-GS115 strain is increased in media with both high and low phosphate concentrations in comparison with control strain. This fits with the data obtained in study, where it was shown that this region may contain a regulatory element involved in* AOX1* promoter repression. The ACP specific activity level of trΔ*SacI*-1-GS115 in medium with high concentration of phosphate did not show statistically significant difference with the one observed in medium with low concentration. This allows proposing that regions of* AOX1* promoter affected by* SacI* truncation may be involved in establishing its regulation by phosphate concentration in medium. ACP activity level of trΔ*NsiI*-1-GS115 strain was significantly lower than the one observed for control strain and did not show statistically significant differences in MPN and MPL media.

### 3.4. Effect of Rapamycin on* AOX1* Promoter Regulation

**Figure 5:**
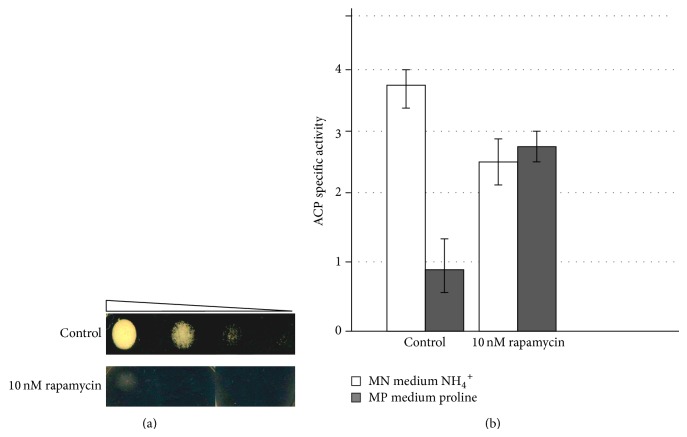
(a) Growth of* P. pastoris* GS115 strain on YPD medium (control) and on medium containing 10 nM rapamycin. (b) Effect of rapamycin treatment on ACP specific activity of* P. pastoris* tr2-1-GS115 strain grown in media with different nitrogen sources (MN with ammonium sulphate and MP with proline).

Kinase inhibitors can provide crucial information about these regulation pathways. In this study we analyzed effect of rapamycin on* P. pastoris* growth and on regulation of* AOX1* gene. To determine active concentrations of rapamycin 10-fold dilutions of* P. pastoris* GS115 culture were placed on YPD medium with different concentrations of rapamycin. It was found that 10 nM concentration is more than enough to inhibit cell growth (Figure 5(a)). These results totally fit with ones used for* S. cerevisiae* [28].

On the next step rapamycin effect on* AOX1* regulation by nitrogen source was investigated. Cultivation was done in two stages due to the fact that rapamycin inhibits* P. pastoris* growth. On the first stage cells of* P. pastoris* tr2-1-GS115 strain were grown for 40 hours to the stationary phase in MPN and MPP media containing 1% glycerol as a carbon source to obtain biomass. After that cultures were centrifuged and transferred into MPN and MPP media containing 1% methanol for induction of* AOX1* promoter and 10 nM rapamycin. ACP specific activity was measured after 40 hours of induction (Figure 5(b)). Addition of rapamycin to the media completely removed repression of* AOX1* promoter in medium with proline.

## 4. Discussion

We show here that expression of main genes involved in methanol utilization (e.g.,* AOX1*,* AOX2*,* PpDAK*,* PpDHAS*,* PpFLD*, and* PpFGH*) and peroxisomal genes (*PpPEX1*,* PpPEX5*,* PpPEX8*, and* PpPEX14*) is tightly regulated depending on nitrogen source. This regulation is established on transcriptional level. In medium with ammonium sulphate expression levels of studied genes were notably higher than in medium with proline as a nitrogen source.

Deletions in* AOX1* promoter affected its activity but did not change promoter regulation by nitrogen source. A deletion in C region of promoter (from −205 to −45) leads to such loss of activity that we were not able to retrieve statistically significant results. This region contains one of binding sites for Mxr1 protein and is essential for induction of* AOX1* gene in medium with methanol [27, 29, 30]. It can be proposed that regulation of* AOX1* promoter is established not by binding a separate transcriptional factor, involved in nitrogen regulation but on the higher level by modulation of Mxr1p activity. Our results obtained from experiment with ACP reporter systems using* AOX1* and* AOX2* promoter may serve as an indirect proof of this proposal. Although the promoter regions of these genes are absolutely different, they are regulated by nitrogen source in the same manner.

Rapamycin treatment completely removed regulation of* AOX1* promoter by nitrogen source, which implies that Tor-kinase plays a key role in establishing this regulation. Addition of rapamycin slightly decreased* AOX1* promoter activity levels when cells were grown in medium with ammonium sulphate, while rapamycin treatment of cells grown in medium with proline increased* PAOX1* activity to the level compared with the one observed in medium with ammonium sulphate. It may be proposed that* AOX1* gene expression is regulated depending on a nitrogen source by a repression mechanism. Recently a 14-3-3 protein that mediates Mxr1p activity and inhibits the expression of genes involved in methanol utilization was found in* P. pastoris* [31, 32]. This protein shows similarity to* S. cerevisiae* Bmh1p, which is involved in regulation of exocytosis, vesicle transport, Ras/MAPK, and rapamycin-sensitive signaling [33].

**Figure 6:**
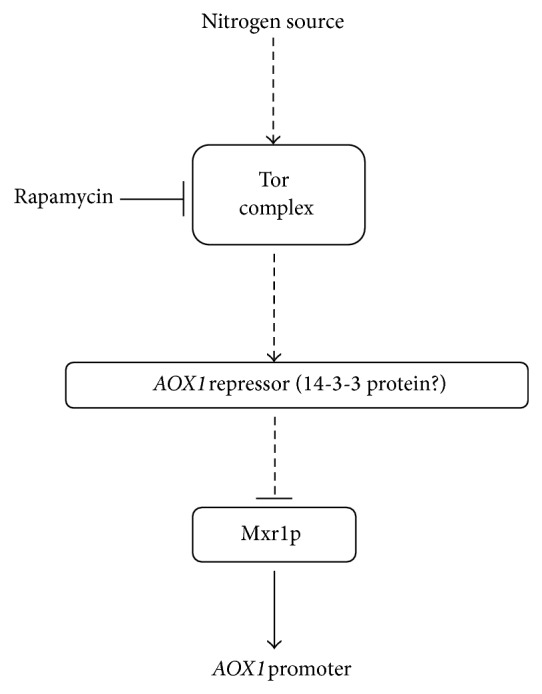
Proposed model of* AOX1* gene regulation by nitrogen source. Activity of* AOX1* promoter is modified depending on nitrogen source via Tor-signaling pathway. Proximate signal transduction is provided by proteins, involved in regulation of* AOX1* gene by carbon source.

A model based on our observations is presented in Figure 6.* P. pastoris* cells discriminate the type of nitrogen source in the medium and modify gene expression via Tor-signalling pathway. Tor-kinase complex modifies activity of transcriptional factors involved in* AOX1* regulation, conceivably 14-3-3 protein that was found to interact with Mxr1p and repress* AOX1*. This mechanism provides maximum induction of* AOX1* promoter in medium with ammonium sulphate and methanol. When proline is used as a nitrogen source* AOX1* activity is repressed to optimum level. It is known that proline can be used by some fungi as a sole carbon source [34]. The ability of* P. pastoris* to use proline as a carbon source is a subject for further investigation.

Apart from nitrogen regulation of genes involved in methanol utilization and peroxisome biogenesis, it was found that these genes also respond to phosphate limitation. Expression levels of* PpDAK*,* PpDAS*,* PpFLD, *and* PpPEX5* genes are increased in media with high concentrations of phosphate. Deletion analysis of* AOX1* promoter has shown that a truncation in* SacI* site changes its regulation by phosphate concentration. Further investigation of this promoter region should be carried out to find if it is involved in establishing of* AOX1* phosphate regulation.

Methylotrophic yeast* P. pastoris* is widely used for production of various heterologous proteins. In biotechnology it is very important to maintain the balance between high levels of heterologous gene expression and cell viability. Decisive understanding of gene regulation mechanisms is essential for reaching this goal. Our result shows that* P. pastoris* have developed a regulation system which coordinates expression of main genes involved in methanol utilization and biogenesis of peroxisomes under different nutrient conditions. This system provides optimal transcriptional levels of* MUT* and* PEX* genes in media with different nitrogen sources or during phosphate limitation.

## 5. Conclusions

Expression levels of main genes involved in methanol utilization (e.g.,* AOX1*,* AOX2*,* PpDAK*,* PpDHAS*,* PpFLD*, and* PpFGH*) and peroxisomal genes (*PpPEX1*,* PpPEX5*,* PpPEX8*, and* PpPEX14*) in* P. pastoris* are notably higher when ammonium sulphate is used as nitrogen source in comparison to proline. Rapamycin treatment removes repression of* AOX1* promoter in medium with proline.

Expression levels of* PpDAK*,* PpDAS*,* PpFLD*, and* PpPEX5* genes are increased in medium with high concentrations of phosphate.

## Figures and Tables

**Table 1:** *P.  pastoris* strains used in this study.

Strain	Genotype	Source
GS115	*his4 *	Invitrogen
1-GS115	*Phox his4 *	[10]
tr2-1-GS115	*Phox PAOX1-PHO5 HIS4 *	[10]
tr3-1-GS115	*phox PAOX2-PHO5 HIS4 *	This study
trΔ*SacI*-1-GS115	*phox PAOX1*Δ*SacI-PHO5 HIS4 *	[10]
trΔ*NsiI*-1-GS115	*phox PAOX1*Δ*NsiI-PHO5 HIS4 *	[10]
trA-1-GS115	*phox PAOX1*Δ*A-PHO5 HIS4 *	This study
trB-1-GS115	*phox PAOX1*Δ*B-PHO5 HIS4 *	This study
trC-1-GS115	*phox PAOX1*Δ*C-PHO5 HIS4 *	This study
trD-1-GS115	*phox PAOX1*Δ*D-PHO5 HIS4 *	This study

**Table 2:** Oligonucleotides used in this study.

Primer	Sequence 5′-3′
PpACT1F	AGTGTTCCCATCGGTCGTAG
PpACT1R	GGTTCATTGGAGCCTCAGTC
PpDHASF	TACGGATGGGAGAGATACGC
PpDHASR	GTGCTTTGGTTTTCCCTTCA
PpDAKF	TGCCCCAGAAATAGACGAAG
PpDAKR	TTCACCACAATCACCATCTCC
PpPEX5F	CGGAGGAGGCAGTAGAGG
PpPEX5R	AAATGCTCTCTTTAGGGTCTCG
PpFLDF	GAATCTTGCCACAAGGGTTG
PpFLDR	TGCTTTGTTGATGTTGTCCAG
PpFGHF	ATCTCCAACCCCACTAAAGC
PpFGHR	CAACGTGAATCAAAATGCTG
PpPEX8F	TTATGATTTGAATGCCCTCGTC
PpPEX8R	CGGGGTTGTTGTTAAGTAGCTG
PpPEX14F	AATGGTTCGTCCTCAGTTGC
PpPEX14R	TAAAGCCCAAACGAAACACC
PpPEX1F	CGGACAAGGAAGCAAGAAAG
PpPEX1R	TCCCCGACTGAACCATTTAG
ACT1probe	CACCACACCTTCTACAACGAGTTGCGT
DHASprobe	GAGAAGATTGGTGAGAAGGTTGCTG
DAKprobe	TATGCTGACCTTCTGGAGTCTGGT
PEX5probe	GGGCAGATATAAAGAGGCTGTTGAAC
FLDprobe	ATCTCTACCCGTCCTTTCCAGTTGG
PpFGHprobe	GACTCAGTACGACCCAACCGAATTG
PpPEX8probe	TGTGCCTTGGATTACGTTGAAGATG
PpPEX14probe	GCCATCTCCTCCTCCTTTTCCTG
PpPEX1probe	GCTGTTGAAACGGAAGGTTATTTACC
PAOX2F	TTCGACGTCCTGCCCTCTCC
PAOX2R	TTCGGATCCTTTTCTCAGTTGATTTG
PAOXFlaF	ACCTGACGTCTAAGAAACCAT
PAOXFlaR	GCGCGGATCCTTCGAATAATT
delAF	AACGCAAATAGCCTAACG
delAR	TTAGGCTATTTGCGTTTCG
delBF	GGAATACTGTTCTAACC
delBR	GAACAGTATTCCCAC
delCF	GCCTAACGACAACTTGAG
delCR	AAGTTGTCGTTAGGCTATCAG
delDR	GGATCCAGTCGC
